# Coal and Rock Hardness Identification Based on EEMD and Multi-Scale Permutation Entropy

**DOI:** 10.3390/e23091113

**Published:** 2021-08-27

**Authors:** Tao Liu, Chao Lu, Qingyun Liu, Yiwen Zha

**Affiliations:** 1 AnHui Province Key Laboratory of Special Heavy Load Robot, Ma’anshan 243032, China; lt_ahut@ahut.edu.cn (T.L.); luchao@ahut.edu.cn (C.L.); zhayiwen@ahut.edu.cn (Y.Z.); 2 School of Mechanical Engineering, Anhui University of Technology, Ma’anshan 243032, China

**Keywords:** coal-rock hardness identification, current signal, EEMD, MPE, Adaboost-BP

## Abstract

This study offers an efficient hardness identification approach to address the problem of poor real-time performance and accuracy in coal and rock hardness detection. To begin, Ensemble Empirical Mode Decomposition (EEMD) was performed on the current signal of the cutting motor to obtain a number of Intrinsic Mode Functions (IMFs). Further, the target signal was selected among the IMFs to reconstruct the current signal according to the energy density and correlation coefficient criteria. After that, the Multi-scale Permutation Entropy (MPE) of the reconstructed signal was trained by the Adaboost improved Back Propagation (BP) neural network, in order to establish the hardness recognition model. Finally, the cutting arm’s swing speed and the cutting head’s rotation speed were adjusted based on the coal and rock hardness. The simulation results indicated that using the energy density and correlation criterion to reconstruct the signal can successfully filter out noise interference. Compared to the BP model, the relative root-mean-square error of the Adaboost-BP model decreased by 0.0633, and the prediction results were more accurate. Additionally, the speed control strategy based on coal and rock hardness can ensure the efficient cutting of the roadheader.

## 1. Introduction

In coal mining operations, the dentification of coal rock hardness is a prerequisite for high-efficiency cutting [1,2]. Due to the complex and variable physical properties of coal and rock, density and hardness are constantly changing and exceedingly unpredictable. Because of the fluctuating loads on the cutting head, the cutting motor’s output power is unstable, and it often performs in an overload or underload state. Therefore, accurate coal hardness detection can assure high coal mining efficiency [3,4]. In recent years, recognition methods have mostly focused on coal-rock image analysis [5,6], multi-sensor information fusion [7,8], acoustic signal analysis [9,10], etc. The idea of the image recognition of coal hardness and type is to compare image features of the working face with those in the database [11]. The identification method selects various features, such as gray scale, texture, shape information, and their combination [12], to represent coal and rock traits. Research has been conducted to determine the type of coal and rock by comparing the gray distribution, average gray value [13], and texture characteristics [14] of the images. However, this method does not always extract the rich feature information in the coal image properly, resulting in an unsatisfying final output. To tackle this problem, Si et al. [15] investigated a new method of coal rock recognition based on the deep convolutional neural network (CNN). Multi-source data fusion is the main way to boost the accuracy of coal and rock recognition. The new features of coal and rock recognition are formed by the fusion of diverse signals such as current, vibration, acoustic emission, and infrared in the way of weight [16,17]. Liu et al. [18] developed a vibration feature extraction method based on Hilbert spectrum information entropy. Through the empirical mode decomposition of the vibration signal of the tail arm bracket of the excavator, it was found that the distribution of the Hilbert spectrum of coal caving differs from that of coal gangue caving. Deshmukh [19] demonstrated that most of the interference signals, such as vibration and coal rock fall, are in the range of 20–5000 Hz. Using an ultrasonic signal as the detecting signal, the interference signal can be eliminated by a signal processing method to achieve the identification of coal and rock. Xu et al. [20] proposed a new method to identify cutting patterns through cutting sound signals of a shearer. The energy of the acoustic signal was extracted as a recognition feature by a wavelet packet transform. Through the time domain processing of the acoustic pressure signal of coal falling, it was found that the peak, variance, and kurtosis indexes are sensitive to the working circumstances. A coal and rock feature recognition method based on the time-domain index of acoustic pressure data was proposed by Xue et al. [21]. The latest signal processing methods include Wavelet Transform (WT) [22], Empirical Mode Decomposition (EMD) [23], Ensemble Empirical Mode Decomposition (EEMD) [24], Empirical Wavelet Transform (EWT) [25], and Variational Mode Decomposition (VMD) [26]. To address the shortcomings of Fourier Transform (FT) and Wavelet Transform (WT) signal processing methods in coal and rock recognition, Wei et al. proposed an improved Empirical Variational Mode Decomposition (EVMD) method for ultrasonic echo signal processing [27].

Although the preceding studies have been successful in determining the hardness of coal and rock, there are still flaws in the accuracy and real-time performance. Noise has a significant impact on the signal due to the complicated mining environment of the roadway. Therefore, according to the energy density and correlation coefficient criteria, this paper reconstructed the current signal after EEMD to achieve signal filtering. The multi-scale permutation entropy of the reconstructed signal was identified by the Adaboost improved BP neural network, in order to perform efficient and accurate coal hardness assessment. The new contributions of this study are as follows:

- It was theoretically analyzed that the fluctuation in load torque not only produces an amplitude–frequency modulation of the stator current but also produces phase modulation, which provides a theoretical basis for stator current as a condition for coal hardness identification.
- The EEMD algorithm was used to decompose and reconstruct the stator current signal for highlighting the power frequency characteristics of the stator current. In addition, multi-scale permutation entropy was used to describe the weak changes of current from multiple scales. Finally, the multi-scale permutation entropy was identified by the Adaboost-BP network to improve the accuracy of coal rock hardness identification.
- An adaptive speed control strategy of the roadheader based on coal hardness was proposed to ensure efficient cutting of the roadheader by adjusting the rotational speed of the cutting head and swing speed of the cutting arm.

The rest of the paper is organized in the following manner. Section 2 investigates the relationship between the cutting stator current and coal rock load. In Section 3, EEMD is applied to decompose and reconstruct the current signal according to the energy density and correlation coefficient criteria. In Section 4, the Adaboost-BP network is utilized to train the MPE eigenvalues derived from the reconstructed signal in order to establish a coal and rock hardness recognition model. In Section 5, to realize expeditious identification of coal rock hardness via the above methods, the Adaboost-BP neural network is used to identify the real-time collected current characteristics, and to control the optimized cutting parameters under a certain hardness by a servo valve and converter to achieve efficient and adaptive cutting of the roadheader.

## 2. Relationship between Load and Current

The variation in the load will influence the current value of the motor. The current is proportional to the load torque. The dynamic formula of the motor drive system is [28]:

(1) $$T_{e}=T_{L}+\frac{J}{p_{n}}\frac{d\omega_{m}}{dt}$$

The relationship between the output torque and the load torque of the cutting head is:

(2) $$M_{t}=\sum Z_{i}r_{i}=T_{L}\cdot \mu \cdot \eta$$

where *T_e_* and *T_L_* are the electromagnetic torque and output torque, respectively; *J* is the moment of inertia of the motor; *p_n_* is the pole number of the motor; $\omega_{m}$ is the motor speed; *Z_i_* is the cutting resistance of the *i*-th pick in the working area, which is related to the rock hardness *P_k_*; *r_i_* is the cutting radius of the *i*-th pick in the working area; μ is the reduction ratio of the reducer; and η is the transmission efficiency of the reducer.

It can be seen from Equation (1) that *T_L_* and $\omega_{m}$ are constant when the motor is stable. If the load torque *M* of the cutting head induces a periodic fluctuation *T_s_* in the output torque *T_L_*, the final steady state of the motor is in dynamic equilibrium. Corresponding fluctuations also occur in electromagnetic torque and motor speed [29]. Therefore, Fourier series expansion of each fluctuation component is carried out:

(3) $$\{\begin{array}{c} T_{L}=T_{L0}+\sum_{i}T_{Li}\cos\left(2\pi f_{i}t+\varphi_{Li}\right)\\ T_{e}=T_{e0}+\sum_{i}T_{ei}\cos\left(2\pi f_{i}t+\varphi_{ei}\right)\\ \omega_{m}=\omega_{m0}+\sum_{i}\omega_{mi}\cos\left(2\pi f_{i}t+\varphi_{mi}\right) \end{array}$$

where *T_L_*_0_, *T_e_*_0_, and $\omega_{m0}$ are the average values of output torque, electromagnetic torque, and motor speed, respectively; *f_i_* is the frequency corresponding to the periodic fluctuation component; *T_Li_*, *T_ei_*, and $\omega_{mi}$ correspond to the amplitude of output torque, electromagnetic torque, and motor speed fluctuation. $\varphi_{Li}$, $\varphi_{ei}$, and $\varphi_{mi}$ correspond to the phase of the output torque, electromagnetic torque, and motor speed fluctuation.

When the motor reaches a steady state, the direct current components and cosine component, respectively, match the following:

(4) $$\{\begin{array}{c} T_{e0}=T_{L0}\\ T_{es}-T_{Ls}=J\frac{d\omega_{ms}}{dt} \end{array}$$

where *T_Ls_*, *T_es_*, and $\omega_{ms}$ are periodic fluctuations in each variable.

The electromagnetic torque equation of the motor with id = 0 vector control mode is:

(5) $$T_{e}=k_{t}i_{q}$$

According to Equations (3) and (5), *i_q_* is the direct current components plus the cosine component of the corresponding frequency:

(6) $$i_{q}=i_{q0}+\sum_{i}i_{qi}\cos\left(2\pi f_{i}t+\varphi_{qi}\right)$$

where *i_q_*_0_ and *i_qi_* denote the average value of the q-axis current and the amplitude of the fluctuation component, respectively; $\varphi_{qi}$ is the phase.

Transforming the current in the *d-q* coordinate system to the *a-b-c* three-phase coordinate system, the following is obtained:

(7) $$\{\begin{array}{c} i_{a}=-i_{q}\sin(\theta )\\ i_{b}=-i_{q}\sin(\theta -120^{\circ })\\ i_{c}=-i_{q}\sin(\theta +120^{\circ }) \end{array}$$

The rotor position information of the motor is:

(8) $$\theta =\theta_{0}+2\pi f_{e0}t+\theta_{tz}$$

where $\theta_{0}$ is the initial position of the rotor, *f_e_*_0_ is the fundamental frequency of the motor current, and $\theta_{tz}$ is the modulation component corresponding to the angular velocity fluctuation.

The expression of a-phase current can be obtained by Equations (6)–(8):

(9) $$\begin{array}{l} i_{a}=i_{q0}\cos\left(2\pi f_{e}t+\theta_{0}+\theta_{tz}+\frac{\pi }{2}\right)+\\ \frac{1}{2}\sum_{i}i_{qi}[\cos\left(2\pi \left(f_{e}+f_{i}\right)t+\varphi_{qi}+\theta_{0}+\theta_{tz}+\frac{\pi }{2}\right)+\\ \cos\left(2\pi \left(f_{e}-f_{i}\right)t-\varphi_{qi}+\theta_{0}+\theta_{tz}+\frac{\pi }{2}\right)] \end{array}$$

According to Equation (9), the periodic fluctuations in the load torque are reflected in the stator current as amplitude modulation and phase modulation. The fluctuation in the load torque impacts not only the current value, but also the motor current signal in the frequency domain.

## 3. Decomposition and Reconstruction of Current Signal of Cutting Motor

### 3.1. EEMD Decomposition of Cutting Motor Current Signal

The IMF decomposed by EMD is clearer than the original signal. In addition, the frequency of IMFs decreases with the order, which is beneficial to the subsequent signal processing [30]. However, the EMD method has the mode mixing phenomenon, so this paper used the EEMD algorithm to decompose the stator current. The EEMD method is a step forward from the EMD method, which can decompose any nonlinear and nonstationary signal into the sum of IMFs and a residual value (*r*) [31,32]. The decomposition steps are as follows:

Step 1: Add white noise *n*(*t*) with a mean value of 0 and a constant standard deviation to the original signal *y*(*t*) in order to obtain a noisy signal *y*′(*t*):

(10) $$y^{\prime }\left(t\right)=y\left(t\right)+n\left(t\right)$$

Step 2: Decompose the noise signal y′(*t*) by EMD to obtain a set of denoised IMFs as *c_k_*(*t*) and a residual component *r*(*t*):

(11) $$y^{\prime }\left(t\right)=\sum_{k=1}^{n}c_{k}\left(t\right)+r\left(t\right)$$

where *c_k_*(*t*) is the *k*-th IMF after EMD decomposition of the noise-containing signal, and *n* is the number of IMFs.

Step 3: Repeat steps 1 and 2 *N* times. The total average of the corresponding IMF is derived on the principle that the statistical mean of the unrelated random series is 0. The IMF after EEMD decomposition is *C_k_*(*t*), which eliminates the influence of adding white noise to the real IMF, where *k* = 1, 2, 3, …, *n*.

(12) $$C_{k}\left(t\right)=\frac{1}{N}\sum_{j=1}^{N}c_{kj}\left(t\right)$$

where *c_kj_*(*t*) is the IMF obtained by EMD by adding white noise to the original signal *y*(*t*) for the *j*-th time.

**Figure 1:**
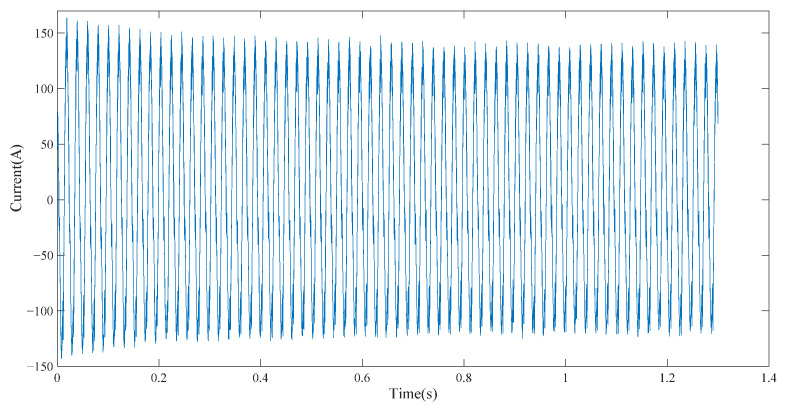
Stator current signal of cutting motor.

In the first section of this paper, a simulation model of the cutting motor was constructed to obtain the stator current signal of the cutting motor, as shown in Figure 1.

**Figure 2:**
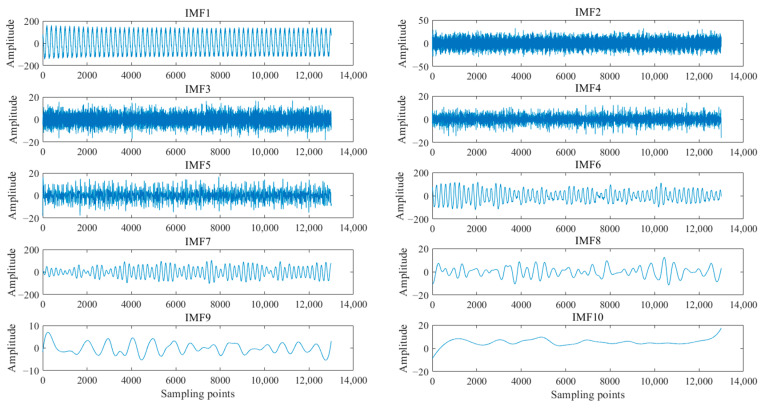
EEMD decomposition results of current signal.

The current signal of the cutting motor was decomposed by the EEMD method. The current signal was decomposed into 10 IMFs with high- to low-frequency distributions, as shown in Figure 2. IMF1 is the original signal and IMF10 is the residual component *r*. The motor stator current signal contains abundant characteristic information of the motor drive system. The amplitude variation of the stator current mainly occurred in the power frequency region. Combined with the 50 Hz power frequency characteristics of the current signal, it can be considered that the IMF component that has a strong correlation with the original current signal contained a strong 50 Hz signal. The decomposed IMF components were reconstructed by the correlation coefficient and energy density to further highlight the power frequency characteristics.

### 3.2. Signal Reconstruction Principle Based on Energy Density and Correlation Coefficient Criterion

For signals mixed with random noise, the high-frequency IMF component is generally noise after decomposition, but low-frequency disturbance is also mixed in the low-frequency portion. Simple frequency analysis cannot eradicate noise, due to complicated work environments of the roadheader [33]. To cope with this problem, this paper selected the IMF component from the energy density and correlation coefficient angle. The steps are as follows:

Step 1: Decompose the current signal of the cutting motor by EEMD to obtain *N* IMF components for energy analysis, and calculate the energy density of each IMF:

(13) $$E_{k}=\frac{1}{K}{\int \left|C_{k}\left(t\right)\right|}^{2}dt=\frac{1}{K}\sum_{d=1}^{K}[x_{k}\left(d\right){]}^{2}$$

where *E_k_* is the energy density of the *k*-th IMF component, *K* is the length of IMF, and *x_k_*(*d*) is the amplitude of the *k*-th IMF component.

Step 2: Analyze the correlation between the IMF component and the current signal of the cutting motor. The mathematical expression is:

(14) $$R_{k}=\frac{E\left[\left(C_{k}-\mu_{k}\right)\left(y-\mu \right)\right]}{\sigma_{k}\sigma }$$

where *R_k_* denotes the mathematical expectation, $\mu_{k}$ is the mean value of IMF components, μ is the mean value of the original current signal *y*, $\sigma_{k}$ is the standard deviation of the IMF component, and σ is the standard deviation of the original current signal *y*.

**Figure 3:**
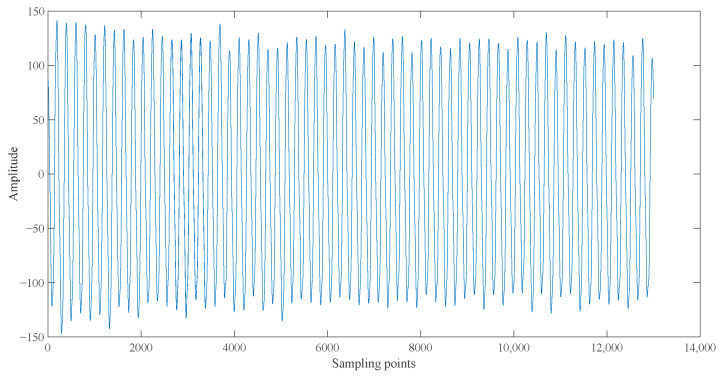
Waveform of reconstructed signal.

The energy density and correlation of IMF components decomposed by EEMD were analyzed. The statistical results of energy density and correlation are shown in Table 1 and Table 2:

According to the statistics, the energy densities of IMF2, IMF6, and IMF7 were all greater than 0.3, which significantly exceeded other-order IMFs. According to the correlation coefficient criterion [34], IMF components of order 2, 3, 4, 5, 8, and 9 with a correlation coefficient less than 0.2 are not correlated with the original current signal. IMF6 and IMF7 were selected as the target signal, and the current signal was reconstructed by superimposing them, as shown in Figure 3. Compared to the original current signal, the reconstructed current signal was smoother, which effectively filters out the high-frequency noise and low-frequency disturbances in the original signal.

## 4. Coal Hardness Identification

### 4.1. Calculation of Multi-Scale Permutation Entropy

The permutation entropy algorithm can effectively enlarge the weak change in time series, and multi-scale permutation entropy can describe the characteristics of time series from multiple scales. In this paper, the multi-scale permutation entropy of stator current was used as the identification feature to accurately discern the hardness of coal rock under complex working conditions. Multi-scale permutation entropy coarse-grains the signal, calculates the permutation entropy of the coarse-grained fragments, and realizes the signal description in multiple dimensions [35]. Coarse-graining the time series *X* = {*x_e_*, *e* = 1, 2, 3…, *F*} with the length of *N* to obtain the coarse-grained sequence $y_{j}^{\left(s\right)}$, the expression is:

(15) $$y_{j}^{\left(s\right)}=\frac{1}{s}\sum_{e=\left(j-1\right)s+1}^{js}x_{e},\;j=1,2,3\cdots ,\left[F/s\right]$$

where *s* is the scale factor and *s* = 1, 2, … [*F*/*s*] denotes rounding *F*/*s*. When *s* = 1, the coarse-grained sequence is the original sequence.

**Figure 4:**
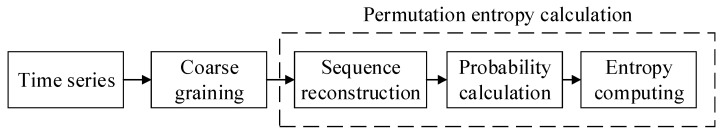
Multi-scale permutation entropy calculation process.

The multi-scale permutation entropy can be obtained by calculating the permutation entropy of each coarse-grained sequence. Figure 4 depicts the calculation process of the multi-scale permutation entropy.

The selection of scale factor is critical in coarse graining. If the scale factor *s* is too small, the characteristics of the signal cannot be retrieved to their full potential. If the value of *s* is too large, the discrepancy between the signals may be erased [36]. Therefore, the particle swarm optimization algorithm was employed in this paper to optimize the scale factor *s*. Skewness represents the degree of probability density a nonnormally distributed random sequence deviates from the normal distribution, which is a digital property of the degree of asymmetry of a statistical data distribution.

The permutation entropy under all scales of the time series *X* = {*x_e_*, *e* = 1, 2, 3…, *F*} is composed of a sequence *H_p_*(*X*) = {*H_p_*(1), *H_p_*(2), …, *H_p_*(*s*)}, and skewness *Ske* can be expressed as:

(16) $$Ske={E{\left[H_{p}\left(X\right)-\bar{H_{p}\left(X\right)}\right]}^{3}/\left[H_{p}^{d}\left(X\right)\right]}^{3}$$

where $\bar{H_{p}\left(X\right)}$ is the mean value of the sequence *H_p_*(*X*), and $H_{p}^{d}\left(X\right)$ is the standard deviation of the sequence *H_p_*(*X*).

The objective function is:

(17) $$F\left(X\right)=Ske^{2}$$

**Figure 5:**
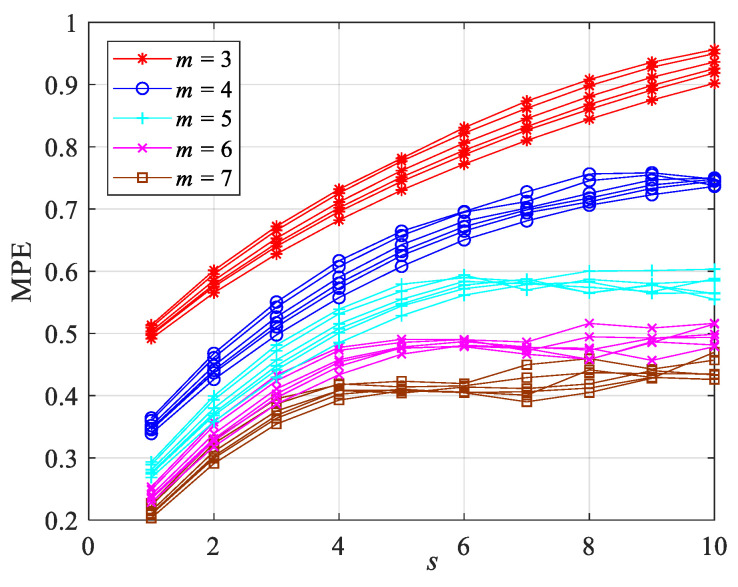
MPE distribution of different coal and rock hardness under different embedding dimensions.

The scale factor *s* = 10 of the multiscale permutation entropy algorithm is obtained by optimizing the skewness of the reconstructed current signal. The embedding dimension *m* is the main parameter of the permutation entropy algorithm. The embedding dimension *m* determines the number of states m!, and the value of permutation entropy is highly dependent on the choice of *m* [37]. If the value of the embedding dimension *m* is too small, the mutation detection performance of the algorithm is degraded in the process of calculating the multiscale permutation entropy. If the embedding dimension *m* is too large, the entropy value will not reflect the subtle changes in the time series. Figure 5 indicates the variation in the MPE of different coal hardness *P_k_* with the scale factor when the embedding dimension is 3–7.

Figure 5 indicates that when the embedding dimension *m* = 4, 5, 6, 7, the MPE values of each scale factor under various hardness values have more overlap, and the MPE struggles to distinguish between them. When the embedding dimension *m* = 3, the MPE value under various hardness values may be clearly differentiated. The MPE value decreases with the coal hardness. The delay time *t* = 5, *m* = 3, and *s* = 10 were determined through the aforementioned research and analysis. The sliding window method was employed for multi-scale entropy extraction to enhance the real-time performance of coal and rock recognition. The rotation of the cutting head was about 1.3 s. The sudden change in coal hardness resulted in a current change of about a 1/8 cycle. A window width of 250 ms, a window increment of 50 ms, and a window overlap rate of 80% were chosen. Table 3 indicates the average entropy values of the cutting current under different coal hardness values. Table 3 indicates that the average MPE varies greatly depending on coal hardness, and that the change trend of the MPE value with the scale factor is the same. MPE can be employed as an essential characteristic parameter of coal hardness through the above analysis.

### 4.2. Adaboost Improved BP Neural Network-Based Coal Hardness Estimation Algorithm

The idea of the Adaboost algorithm is to combine the outputs of multiple weak learners to generate effective predictions. The BP network is prone to falling into the local optimal solution, has a poor prediction ability, and has a limited generalization ability [38,39]. The BP neural network is regarded as a weak learner, and the Adaboost algorithm was applied to merge the output results of multiple BP weak learners to output more accurate prediction results. The process is as follows:

Step 1: Data selection and network initialization. *h* groups of training data *T* = {(*x*_1_,*y*_1_), (*x*_2_,*y*_2_), …, (*x_h_*,*y_h_*)} are randomly selected from the sample space to initialize the distribution weight of the test data *D_i_* = 1/*h* (*i* = 1, 2…, *h*). The neural network structure is determined according to the sample input and output dimensions, and the BP neural network weights and thresholds are initialized.

Step 2: Weak learner prediction. The BP neural network is trained with the training data. The output of the training data is predicted to obtain the prediction error *e_t_* of the prediction sequence *g*(*t*). The calculation of the error *e_t_* is:

(18) $$e_{t}=\sum_{i}D_{i}\left(i\right)\;\;\;i=1,2,3,\ldots ,h\;\left(g_{t}(x_{i})\neq y_{i}\right)$$

Step 3: Calculate the weight *C_t_* of the *t*-th BP learner.

(19) $$C_{t}=\frac{1}{2}\ln(\frac{1-e_{t}}{e_{t}})$$

Step 4: Modify the weight *D_t_*_+1_(*i*) of the next round of training samples according to the weight *C_t_* of weak learning.

(20) $$D_{t+1}(i)=\frac{D_{t}(i)}{B_{t}}\exp[-C_{t}y_{i}g_{t}(x_{i})]$$

where *B_t_* is the normalization factor.

Step 5: Integrate strong learners. After *T* iterations of training, *T* groups of weak learner functions *f*(*x*) and their weight vectors are generated and integrated into a strong learner function by weighted manner.

(21) $$F(x)=sign\left[\sum_{t=1}^{T}C_{t}f_{t}(x)\right]$$

where *F*(*x*) is the strong learner function and *f_t_*(*x*) is the *t*-th weak learner function.

**Figure 6:**
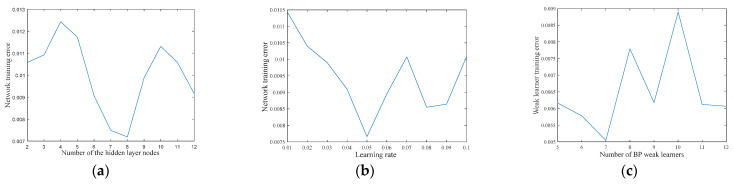
The influence of network parameters on training error: (**a**) number of hidden layer nodes, (**b**) learning rate, and (**c**) number of BP weak learners.

The input layer of the BP neural network is 10 and the output layer is 1. Figure 6 shows the influence of the hidden layer, learning rate, and the number of BP weak learners in the Adaboost strong learner model on network training error. Figure 6 indicates that when the number of hidden layer nodes is 8, the training error is 0.007188; when the learning rate is 0.05, the training error is 0.0076589; when the number of weak learners is 7, the training error is 0.0036743. The number of hidden layer nodes, the learning rate, and the number of weak learners of the BP network are determined to be 8, 0.05, and 7.

**Figure 7:**
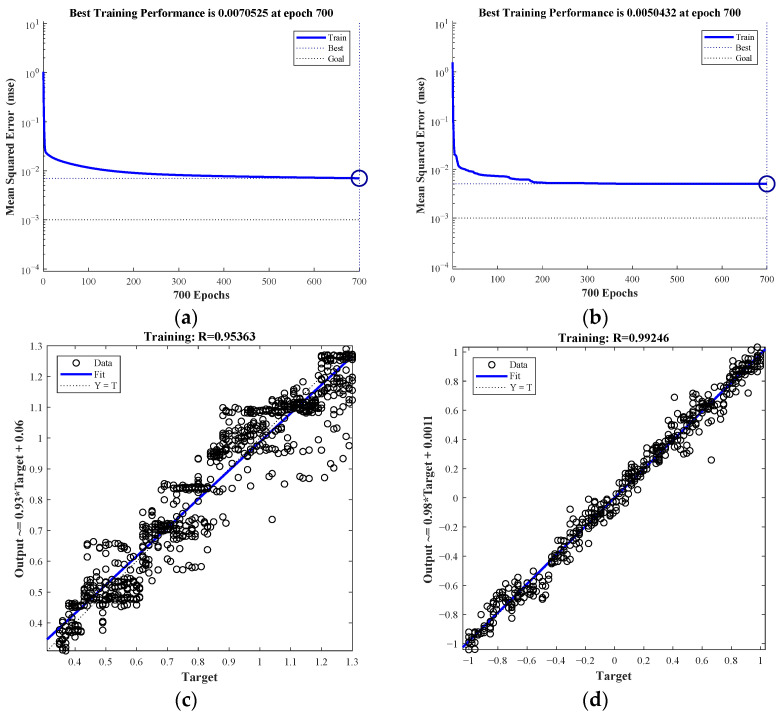
BP and Adaboost-BP model training status diagram: (**a**) error drop-down curve of BP model, (**b**) error drop-down curve of Adaboost-BP, (**c**) regression state of BP model, and (**d**) regression state of Adaboost-BP.

Figure 7 shows the training status of the BP model and Adaboost-BP model. Figure 7a indicates that the BP model iterates 150 times with an error of 10^−2^ magnitude, while the Adaboost-BP model iterates 20 steps with an error of 10^−2^ magnitude. Therefore, the Adaboost-BP model outperforms the BP model in terms of convergence speed and network training error. The regression coefficient R indicates how well the network fits the data. Figure 7c,d demonstrate that the Adaboost-BP model outperforms the BP model in fitting data samples.

The relative root-mean-square error (RMSE) was applied to determine the dispersion degree of prediction results, and the coal hardness prediction model was evaluated.

(22) $$\mathrm{RMSE}=\sqrt{\frac{\sum_{i=1}^{T}{\left[\overset{⌢}{y}(i)-y(i)\right]}^{2}}{\sum_{i=1}^{T}y^{2}(i)}}$$

where $\overset{⌢}{y}(i)$ is the estimated hardness of coal and rock, *y*(*i*) is the actual hardness value of coal rock, and *T* is the sample size.

Quantitative analysis was performed on the estimation effects of the six types of coal and rock hardness. The RMSE results of the BP model and Adaboost-BP model are shown in Table 4. The RMSE value of the Adaboost-BP model was smaller than that of the BP model, and the average value decreased by 0.0633. Therefore, the estimated effect of the Adaboost-BP model was significantly higher than that of the BP model.

**Figure 8:**
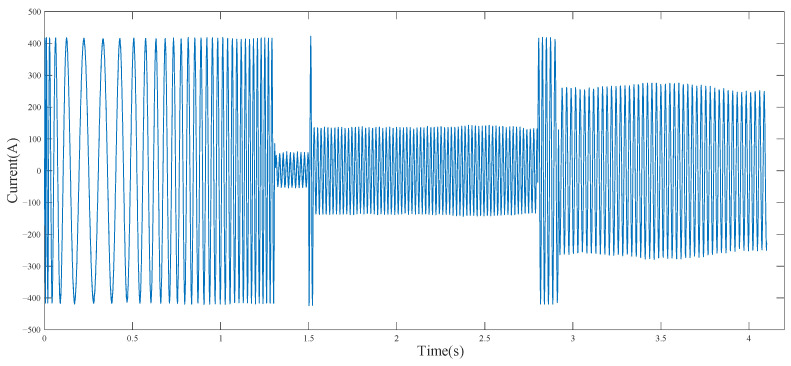
Stator current signal of cutting motor.

**Figure 9:**
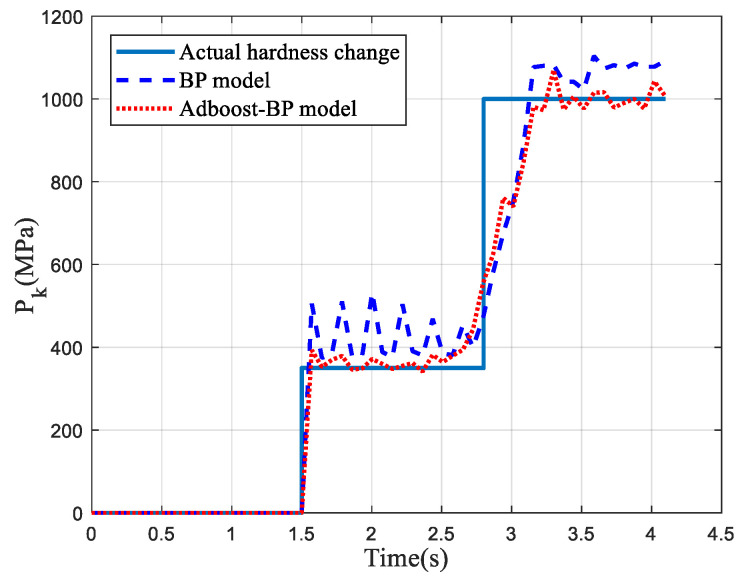
Estimation results of two models.

Figure 8 indicates the stator current of the cutting motor under the change in coal rock hardness from *P_k_* = 350 to *P_k_* = 1000. In Figure 8, the hardness of coal and rock in the starting stage was 0 MPa before 1.5 s and changed from 350 MPa to 1000 MPa after 2.8 s. BP and Adaboost-BP network were used to identify the multi-scale permutation entropy of current and predict the hardness of coal. The result is shown in Figure 9.

In Figure 9, the coal hardness estimated by the Adaboost-BP model was more consistent with the changing trend of the actual coal hardness. Therefore, the coal and rock hardness estimation model based on the Adaboost-BP neural network was superior to the BP model in coal hardness identification.

## 5. Control and Simulation

### 5.1. Adaptive Speed Control Based on Coal Rock Hardness Change

The purpose of hardness identification is to realize cutting arm and cutting head speed regulation. The speed regulation method of constant power ensures that the cutting motor runs at a constant power but its cutting efficiency is not the highest. The cutting motor is often overloaded or underloaded due to the load fluctuation on the cutting head. In order to improve the cutting performance under different coal hardness values, the fluctuation coefficient of the cutting head load $K_{R_{a}}$, $K_{R_{b}}$, $K_{R_{c}}$, and $K_{M_{t}}$ and the specific energy consumption *H_W_* were used as optimization targets, the swing (*v*) and rotation (*n*) speeds were selected as the optimized variables, and the cutting motor power and speed range were used as constraints. For optimization methods and parameters, please refer to the literature [40], and the optimization results are shown in Table 5 and Table 6.

**Figure 10:**
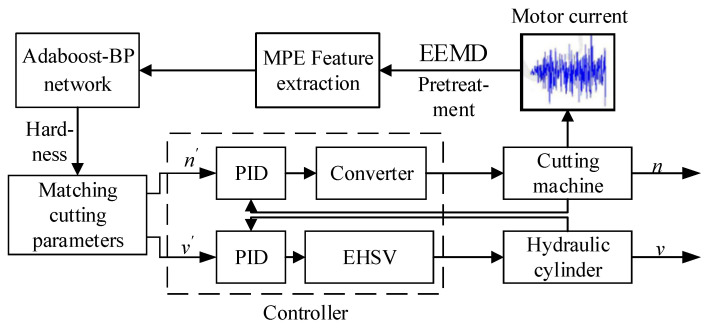
Adaptive variable speed cutting control strategy.

It can be seen from Table 5 that the cutting performance significantly improved after optimization. In order to ensure that the roadheader was in an efficient cutting state under different coal rock hardness values, an adaptive speed control strategy based on coal rock hardness was proposed, as shown in Figure 10. EEMD and reconstruction were conducted on the real-time collection of cutting motor current. The hardness of coal was evaluated by the neural network, and the optimal cutting parameters under this hardness were matched according to the optimization results. The matching cutting parameters were employed as control signals. The swing and rotation speeds were controlled through the electro-hydraulic servo valve (EHSV) and the converter, to ensure that the cutting performance of the roadheader was always in the ideal cutting state.

### 5.2. Adaptive Speed Control Simulation

**Figure 11:**
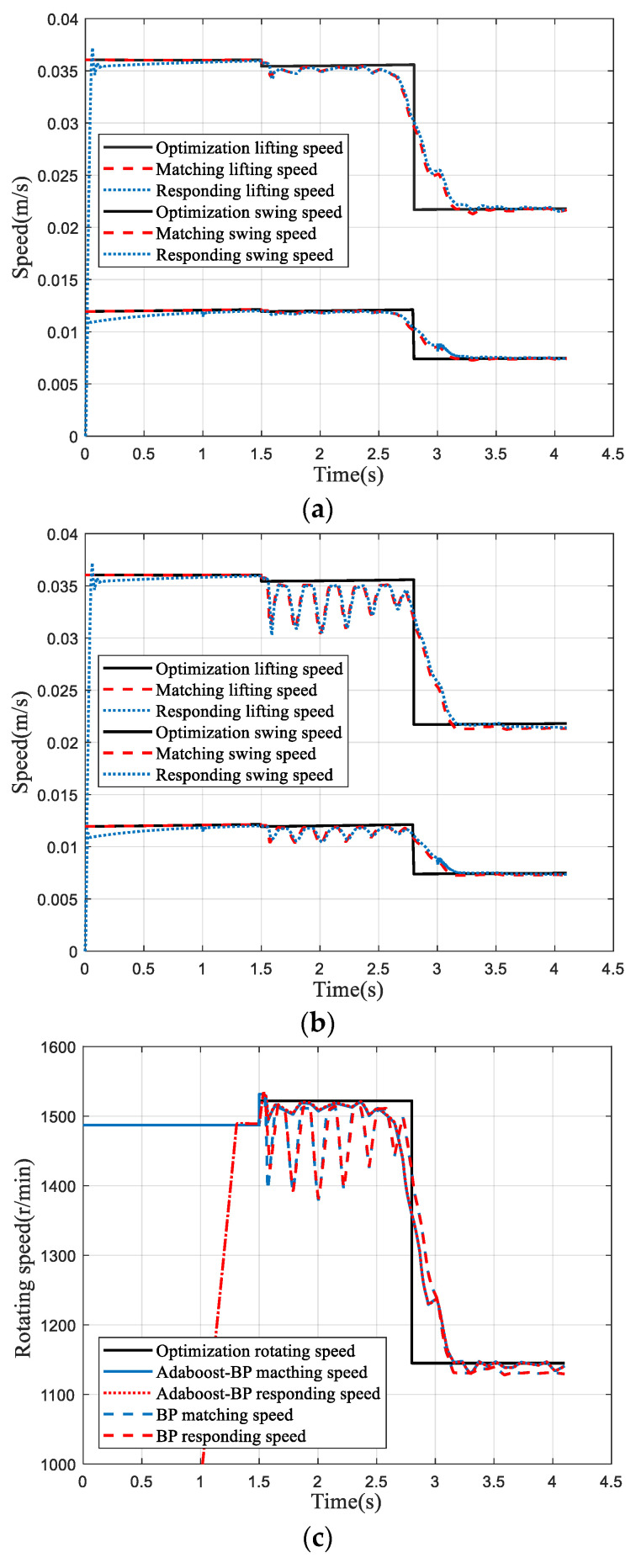
Adaptive speed control simulation curve: (**a**) speed regulation curve of hydraulic system under Adaboost-BP model, (**b**) speed regulation curve of hydraulic system under BP model, and (**c**) cutting motor speed control curve.

Matlab and AMESim were applied to simulate the cutting speed regulation system of the roadheader to verify the feasibility of the adaptive cutting speed regulation strategy of the roadheader based on coal rock hardness. According to Table 6, the speed of the cutting head was reduced from 48.28 r/min to 36.9 r/min, and the swing velocity of the cantilever decreased from 2.43 m/min to 1.5 m/min, when the hardness of coal changed from *P_k_* = 350 to *P_k_* = 1000. The speed of the cutting motor was controlled by Space Vector Pulse Width Modulation (SVPWM). The transmission ratio of the cutting head was *n*_1_ = 32. The hydraulic system adopted electro-hydraulic servo valve control. Comparing the speed control results under the Adaboost-BP and BP prediction models, the simulation results are shown in Figure 11.

Figure 11 indicates that for the cutting motor or the hydraulic system, the speed regulation results based on the Adaboost-BP estimation model were basically consistent with the optimized results. The speed control results based on the BP estimation model fluctuated and deviated significantly from the optimization results. The adaptive speed control based on the Adaboost-BP estimation model can ensure that the roadheader is in the optimal cutting state.

## 6. Conclusions

In order to enhance the accuracy and efficiency of coal rock hardness identification, the multi-scale permutation entropy of stator current was determined as the identification trait by analyzing the relationship between stator current and load. The Adaboost-BP neural network was utilized to train the characteristics to construct the coal rock hardness estimation model. The following conclusions can be drawn from the results in this study.

The reconstruction method based on energy density and the correlation coefficient criterion could effectively filter out the noise interference in the signal. The reconstructed stator current signal was extracted by multi-scale permutation entropy after parameter optimization. The results indicated that when the delay time was 5, the embedding dimension was 3, and the scale factor was 10, the multi-scale permutation entropy could better distinguish the hardness of coal and rock.

The Adaboost-BP prediction model was applied to identify the hardness of coal and rock. The Adaboost-BP prediction model proposed in this paper outperformed the BP prediction model in terms of hardness prediction. Simultaneously, the speed regulation method based on coal rock hardness could effectively regulate the speed of the cutting head and the cutting arm.

In this paper, we proposed an efficient hardness identification approach for the detection of coal and rock hardness. However, it should be realized that using the stator current characteristics as the only input signal for identification may be insufficient. It is worth noting that multi-source data fusion technology [41] can overcome this deficiency because this technology can enrich the feature information of the identified object. In addition, the Adaboost-BP neural network has defects such as long training time and difficulty in determining the number of weak learners. The Deep Temporal Convolution Network (DTCN) has a higher efficiency in training samples and data classification [42]. Therefore, combining deep learning technology and multiple data fusion technology to identify the hardness of coal and rock will be interesting work for our future studies.

## Figures and Tables

**Table 1:** IMF component energy density statistics for current signal.

Mean Energy	IMF2	IMF3	IMF4	IMF5	IMF6	IMF7	IMF8	IMF9
*E*(10^−2^)	0.6615	0.1529	0.0097	0.1855	20.49	13.11	0.1281	0.0038

**Table 2:** Statistics of the correlation between the IMF component and the original signal.

Average Correlation Coefficient	IMF2	IMF3	IMF4	IMF5	IMF6	IMF7	IMF8	IMF9
*R*	0.0602	0.0279	0.0387	0.1396	0.9455	0.9079	0.0389	0.0005

**Table 3:** Mean value of multi-scale permutation entropy under different coal hardness values.

*s*	1	2	3	4	5	6	7	8	9	10
*P_k_* = 350	0.5198	0.6122	0.6862	0.7483	0.8011	0.85	0.8896	0.9225	0.9504	0.9708
*P_k_* = 490	0.5175	0.6078	0.6817	0.7448	0.7985	0.8451	0.8848	0.918	0.9434	0.9678
*P_k_* = 650	0.5045	0.587	0.6556	0.7143	0.7656	0.8107	0.8502	0.884	0.9139	0.9388
*P_k_* = 800	0.4993	0.5785	0.6443	0.7009	0.7508	0.7951	0.8343	0.8683	0.8988	0.9235
*P_k_* = 1000	0.4945	0.5708	0.6343	0.6898	0.7389	0.7823	0.821	0.8556	0.8861	0.9122
*P_k_* = 1300	0.4909	0.5652	0.6275	0.6818	0.7299	0.7721	0.8105	0.8441	0.875	0.9013

**Table 4:** RMSE under different coal hardness values.

***P_k_* (MPa)**	350	490	650	800	1000	1300
**BP**	0.2864	0.1275	0.1545	0.0734	0.0486	0.0585
**Adaboost-BP**	0.0707	0.102	0.0955	0.0532	0.0299	0.0285

**Table 5:** Comparison of optimization results for *P_k_* = 350.

*P_k_* = 350 (MPa)	*n* (r/min)	*v* (m/min)	$K_{R_{a}}$	$K_{R_{b}}$	$K_{R_{c}}$	$K_{M_{t}}$	$H_{W}(kW\cdot h/m^{3})$
Before optimization	50	2.5	0.0117	0.0612	0.0236	0.0106	1.017
After optimization	48.28	2.43	0.011	0.0583	0.0215	0.0102	0.9136

**Table 6:** Motion parameters after optimization.

***P_k_*** **(MPa)**	350	490	650	800	1000	1300
***n*** **(r/min)**	48.28	43.96	40.15	38	36.91	34.17
***v*** **(m/min)**	2.43	2.18	1.92	1.7	1.508	1.38

## Data Availability

Not applicable.
